# A Novel Interaction between hScrib and PP1γ Downregulates ERK Signaling and Suppresses Oncogene-Induced Cell Transformation

**DOI:** 10.1371/journal.pone.0053752

**Published:** 2013-01-24

**Authors:** Kazunori Nagasaka, Takayuki Seiki, Aki Yamashita, Paola Massimi, Vanitha Krishna Subbaiah, Miranda Thomas, Christian Kranjec, Kei Kawana, Shunsuke Nakagawa, Tetsu Yano, Yuji Taketani, Tomoyuki Fujii, Shiro Kozuma, Lawrence Banks

**Affiliations:** 1 Department of Obstetrics and Gynecology, Faculty of Medicine, The University of Tokyo, Tokyo, Japan; 2 International Centre for Genetic Engineering and Biotechnology, Area Science Park, Trieste, Italy; 3 Department of Obstetrics and Gynecology, The Teikyo University School of Medicine, Tokyo, Japan; Northwestern University Feinberg School of Medicine, United States of America

## Abstract

Previous studies have shown that the cell polarity regulator hScrib interacts with, and consequently controls, the ERK signaling pathway. This interaction occurs through two well-conserved Kinase Interacting Motifs, which allow hScrib to bind ERK1 directly, resulting in a reduction in the levels of phospho-ERK. This suggests that hScrib might recruit a phosphatase to regulate this signaling pathway. Using a proteomic approach we now show that Protein Phosphatase 1γ (PP1γ) is a major interacting partner of hScrib. This interaction is direct and occurs through a conserved PP1γ interaction motif on the hScrib protein, and this interaction appears to be required for hScrib's ability to downregulate ERK phosphorylation. In addition, hScrib also controls the pattern of PP1γ localization, where loss of hScrib enhances the nuclear translocation of PP1γ. Furthermore, we also show that the ability of hScrib to interact with PP1γ is important for the ability of hScrib to suppress oncogene-induced transformation of primary rodent cells. Taken together, these results demonstrate that hScrib acts as a scaffold to integrate the control of the PP1γ and ERK signaling pathways and explains how disruption of hScrib localisation can contribute towards the development of human malignancy.

## Introduction

The control of cell polarity and the maintenance of tissue architecture are intimately related and are, in part, controlled by a tri-partite macromolecular signaling complex consisting of the Scrib complex, the Par complex and the Crumbs complex [1], [2]. Through a series of antagonistic interactions the components of these three complexes control a variety of downstream signaling pathways that, in turn, directly contribute to the regulation of cell polarity and cell proliferation [3]. It is now clear that the loss of control of these pathways is a common event during the development of diverse human malignancies [1], [4]–[7]. These defects are particularly evident at the later stages of malignant progression, and a variety of studies in both Drosophila and transgenic mice have provided additional supporting evidence of tumour suppressor activity for the various components of these signaling complexes [8]–[11].

The hScrib complex consists of three proteins, hScrib, hDlg1 and Hugl-1/2. In *Drosophila*, loss of either Scrib or Dlg produces imaginal disc overgrowth with invasive characteristics [8] [12], phenotypes that can be functionally complemented by the mammalian equivalents [13]–[15]. More recently Scrib has been implicated in the control of the JNK and ERK signaling cascades, and loss of hScrib appears to enhance the effects of the Ras and Myc oncogenes, and can contribute to mammary tumour development [16]–[21]. Recent studies have also demonstrated that hScrib can interact directly with ERK, and control both ERK activation and its nuclear translocation [19]. However, the physical interaction between ERK and hScrib is not sufficient to explain the inactivation of ERK, since high levels of hScrib appear capable of directly reducing the levels of ERK phosphorylation [19]. Since hScrib has no known phosphatase activity itself, it therefore seemed possible that a protein phosphatase might be recruited by hScrib to fully inactivate the ERK signaling pathway.

Control of ERK activation reflects an exquisite balance between the activities of the activating kinases and the de-activating protein phosphatases. Activated ERK can translocate to the nucleus, where it activates several transcription factors and also phosphorylates cytoplasmic and nuclear kinases [22]–[24]. Since phosphorylation of both the threonine and tyrosine residues of ERK is required for its activation, dephosphorylation of either is sufficient for its inactivation [25]. There are several reports demonstrating that dephosphorylation of active ERK can be achieved by tyrosine-specific phosphatases, by serine/threonine-specific phosphatases or by dual specificity (threonine/tyrosine) protein phosphatases [26]–[29]. One of the important negative regulators of the ERK signaling pathway is PP2A, a member of the PPP family of protein serine/threonine phosphatases which also includes PP1 [30], [31]. However, PP2A is thought to exert its activity mainly upon other activating kinases within the cascade, rather than upon ERK itself [32]–[34]. In addition, recent studies have also shown that hScrib can directly regulate the Akt signaling cascade by recruitment of the protein phosphatase PHLPP1 to the plasma membrane, thereby resulting in de-phosphorylation of Akt [35]. Here, we have used a proteomic approach to extend our investigations into the regulation of the ERK signaling cascade by hScrib. We now show that hScrib interacts with PP1γ, and that this association correlates with the ability of hScrib to downregulate ERK activation. We also provide compelling evidence that hScrib directly contributes to the regulation of PP1γ function by controlling its translocation between the cytoplasm and the nucleus. Thus, loss of hScrib expression results in both ERK activation and aberrant nuclear translocation of PP1γ.

## Materials and Methods

### Cells and treatments

HEK293 (human embryonic kidney cells) and HaCaT (Human keratinocytes) were obtained from ATCC [36], [37]. HEK293, HaCaT and Baby Rat Kidney (BRK) cells were cultured in Dulbecco's modified Eagle's medium (DMEM) supplemented with 10% fetal bovine serum, penicillin-streptomycin (100 U/mL) and glutamine (300 µg/mL) in a humidified 5%CO_2_ incubator. Transfection was carried out using calcium phosphate precipitation as described previously [37] or using Lipofectamine 2000 (Invitrogen) according to the manufacturer's protocol. The depleted Scribble cell lines were generated as described previously [19]. Cell transformation assays were done using BRK cells obtained from 9 day old Wistar rats with a combination of HPV-16 E7 and EJ-ras, plus the appropriate hScrib and PP1γ expression plasmids. Cells were placed under G418 selection for three weeks, and then fixed and stained.

### Plasmids

The wild type pCDNA3-HA-PP1γ was the kind gift of Dr. Wilhelm Krek (Swiss Federal Institute of Technology (ETH) Zurich). The wild type HA-tagged pcDNA hScrib expression plasmid and the truncated mutant pGEX hScrib PDZ1-C, PDZ1-4, S1445A, S1445D, and CT expression plasmids have been described previously [19]. The L1266Y1268→AA mutation (KADA) to doubly change the Leucine (L) and Tyrosine (Y) residues to Alanine (A) in hScrib was done using the QuikChange site-directed mutagenesis kit from Stratagene Cloning Systems (Celbio) according to the manufacturer's instruction. The mutants were confirmed by DNA sequencing. See Figure S1 for a detailed description of the location of the different hScrib mutations.

### Antibodies

The following commercial antibodies were used at the dilution indicated: anti-hScrib goat polyclonal antibody (Santa Cruz, WB 1∶1000), anti-PP1γ goat polyclonal antibody (Santa Cruz, WB 1∶1000), anti-PP1γ sheep polyclonal antibody (Abcam, WB 1∶1000), anti-p44/42 MAPK (Erk1/2) antibody (Cell Signaling Technology, WB 1∶1000), anti-phospho p44/42 MAPK (Erk1/2) (Thr202/Tyr204) antibody (Cell Signaling Technology, WB 1∶1000), anti-HA monoclonal antibody 12CA5 (Roche, WB 1∶500), anti-γ-tubulin monoclonal antibody (Sigma, WB 1∶5000), anti-p84 mouse monoclonal antibody (Abcam, WB 1∶1000), anti-E-Cadherin rabbit polyclonal antibody (Santa Cruz, WB 1∶500), anti-α-tubulin mouse monoclonal antibody (Abcam, WB 1∶1000).

### Immunofluorescence and Microscopy

For immunofluorescence cells were grown on glass coverslips and fixed in 3.7% paraformaldehyde in PBS for 20 mins at room temperature. After washing in PBS the cells were permeabilised in PBS/0.1% Triton for 5 mins, washed extensively in PBS and then incubated with primary antibody diluted in PBS for 1 hour followed by the appropriately conjugated secondary antibodies. Secondary antibodies conjugated to Alexa Fluor 488 or 548 were obtained from Invitrogen. The cells were then washed several times in water and mounted on glass slides. Cells were visualized by using a Zeiss Axiovert 100 M microscope attached to a LSM 510 confocal unit.

### siRNA transfection

HEK293 cells were seeded on 6 cm dishes and transfected using Lipofectamine 2000 (Invitrogen) with control siRNA against Luciferase (siLuc), or siRNA against hScrib and PP1γ sequences (Dharmancon). 48 hours post-transfection cells were harvested and total cells extracts or cell fractionated extracts were then analysed by western blotting.

### Fusion protein purification and in vitro binding assays

GST-tagged fusion proteins were expressed and purified as described previously [19]. Proteins were translated in vitro using the Promega TNT kit and radiolabelled with (^35^S) cysteine or (^35^S) methionine (Perkin Elmer). Equal amounts of in vitro-translated proteins were added to GST fusion proteins bound to glutathione agarose (Sigma) and incubated for 1 hour at 4**°**C. After extensive washing with PBS containing 0.25% NP-40, or as otherwise indicated, the bound proteins were analysed by SDS-PAGE and autoradiography.

### In vitro phosphorylation

Purified GST fusion proteins were incubated with commercially purified ERK1 (Cell Signaling Technology) or PKA (Promega) for 20 mins at 30°C in phosphorylation buffer (0.25 M Tris pH7.5, 1 M MgCl_2_, 3 M NaCl, 0.3 mM aprotinin, 1 mM Pepstatin) or using the kinase buffer supplied by New England Biolabs supplemented with 56 nM (^32^P) γ-ATP (Perkin Elmer) and 10 mM ATP following the manufacturer's instruction. After extensive washing, the phosphorylated proteins were monitored by SDS-PAGE and autoradiography.

### Mass spectrometry analysis

HEK293 cells were transfected with HA-tagged Scrib and after 24 hours the cells were extracted in mass spectrometry lysis buffer (50 mM Hepes pH 7.4, 150 mM NaCl, 50 mM NaF, 1 mM EDTA, 0.25% NP40) and extracts incubated with anti-HA beads (Sigma) for 2–3 hours on a rotating wheel at 4°C. The beads were then extensively washed with PBS, dried and the immunoprecipitated proteins were subjected to proteomic analysis as described previously [38].

### Subcellular Fractionation assays

Differential extraction of HEK 293 cells to obtain cytoplasmic, membrane, cytoskeleton, and nuclear fractions was performed using the Calbiochem ProteoExtract Fractionation Kit according to the manufacturer's instructions. To inhibit phosphatase activity during the preparation of cell lysates, phosphatase inhibitors (1 mM Na_3_VO_4_, 1 mM β-Glycerophosphate, 2.5 mM Sodium Pyrophosphate, 1 mM Sodium Fluoride) were also included.

### Immunoprecipitation and Western blotting

Total cellular extracts were prepared by directly lysing cells from dishes in SDS lysis buffer. Alternatively cells were lysed in either E1A buffer (25 mM HEPES pH 7.0, 0.1% NP-40, 150 mM NaCl, plus protease inhibitor cocktail; Calbiochem) or RIPA buffer (50 mM Tris HCl pH 7.4, 1% NP-40, 150 mM NaCl, 1 mM EDTA, plus protease inhibitor cocktail; Calbiochem) and the cell extracts were analysed by SDS-PAGE and western blotting. For immunoprecipitations, total cell lysates were transferred into a tube of equilibrated EZview Red Anti-HA Affinity Gel beads (Sigma), and incubated for 2 hours at 4°C. Immunoprecipitates were extensively washed four times in lysis buffer and solubilised in SDS-PAGE sample buffer. For western blotting, 0.45 µm nitrocellulose membrane (Schleicher and Schuell) was used and membranes were blocked for 1 hour at 37°C in 10% milk/PBS followed by incubation with the appropriate primary antibody diluted in 10% milk/0.5% Tween 20 for 1 hour. After several washings with PBS 0.5% Tween 20, secondary antibodies conjugated with HRP (DAKO) in 10% milk/0.5% Tween 20 were incubated for 1 hour. Blots were developed using Amersham ECL reagents according to the manufacturer's instructions.

## Results

### PP1γ is a direct binding partner of hScrib

**Figure 1:**
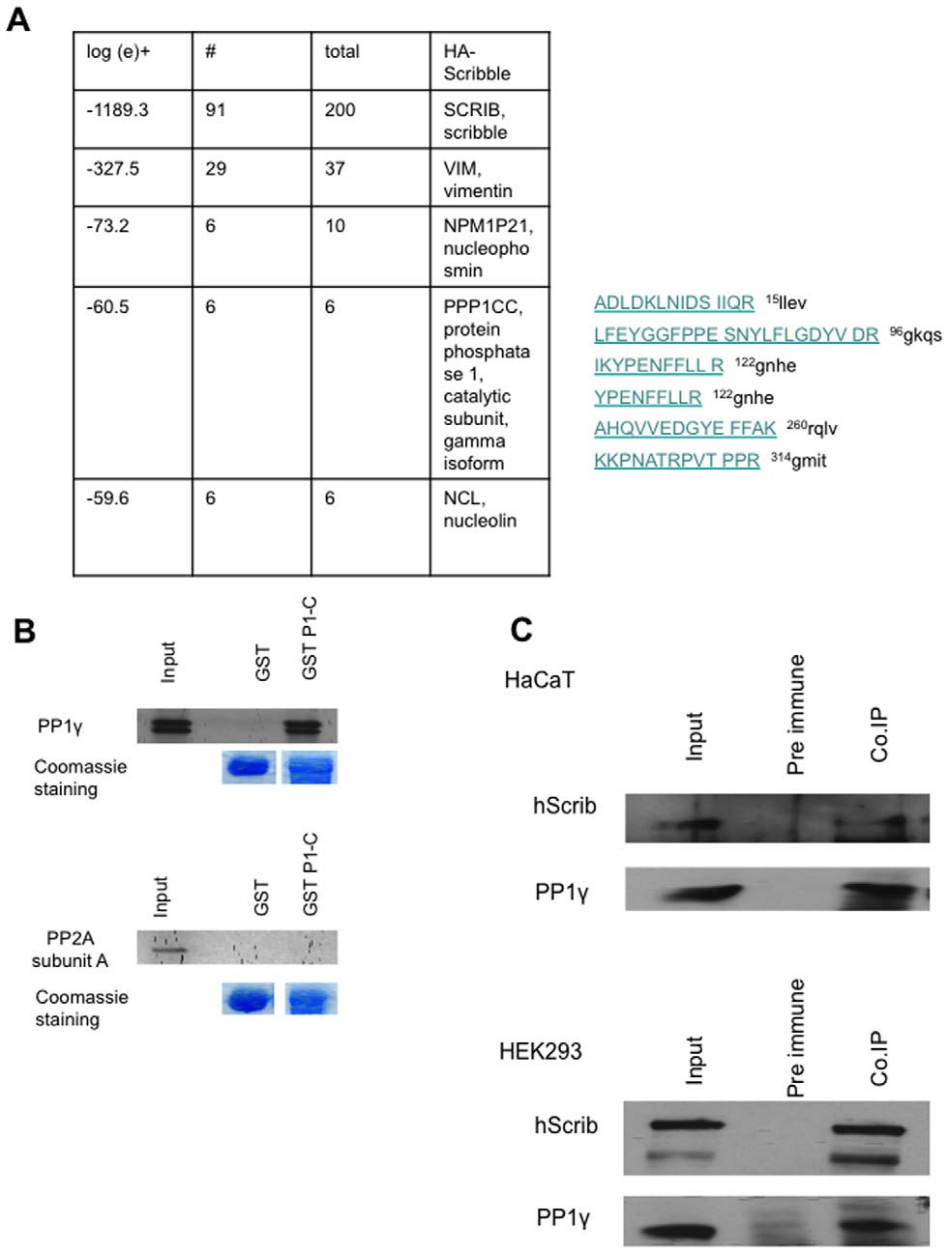
Interaction between hScrib and PP1γ in vivo. A) Results from the mass spectroscopy analysis of hScrib containing immunoprecipitates identified 6 peptides (indicated) corresponding to PP1γ. B) In vitro translated PP1γ (upper panels) and PP2A subunit A (lower panels) were incubated for 1 hour at 4°C with purified GST-hScribP1-C or GST alone immobilized on Glutathione agarose. After extensive washing, the bound proteins were analysed by SDS–PAGE and autoradiography which are shown in each of the upper panels. The gels were rehydrated and stained with Coomassie to show equal levels of GST loading in the respective lower panels. C) Endogenous PP1γ was immunoprecipitated from HaCaT (upper panels) and HEK 293 cells (lower panels), with pre-immune antibody used as control. The immunoprecipitated proteins were then analysed by western blotting using anti-hScrib and anti-PP1γ antibodies.

Based on our previous studies we reasoned that down-regulation of ERK phosphorylation by hScrib might involve the recruitment of a protein phosphatase [19]. To investigate this possibility we performed proteomic analyses to identify additional interacting partners of hScrib. HEK293 cells were transfected with an HA-tagged hScrib expression plasmid and after 24 hours the cells were extracted, and hScrib–bound protein complexes were immunoprecipitated with anti-HA agarose beads and then subjected to mass spectroscopy analysis. Several previously reported interacting partners were identified, including vimentin. However, of the novel interacting partners, the most prominent phosphatase identified was the catalytic subunit of PP1γ (Figure 1A), a major eukaryotic serine/threonine protein phosphatase. To investigate whether hScrib can interact with PP1γ, an in vitro pull-down assay was performed using purified GST-hScrib P1-C fusion protein and in vitro translated radiolabeled PP1γ. For comparison a similar assay was also done using in vitro translated radiolabeled protein phosphatase 2A (PP2A). After extensive washing the bound PP1γ and PP2A were detected by SDS PAGE and autoradiography, and the results in Figure 1B demonstrate strong interaction between hScrib and PP1γ. In contrast, no interaction was observed between hScrib and PP2A, confirming the specificity of the association between hScrib and PP1γ. To determine whether endogenous hScrib and PP1γ could exist in a complex in vivo, immunoprecipitations were performed on cell extracts from HEK293 and HaCaT epithelial cells using anti-PP1γ antibody. Co-immunoprecipitated hScrib was then detected by western blotting, and the results in Figure 1C show a significant degree of co-immunoprecipitation of hScrib with PP1γ in both cell lines. Taken together, these results demonstrate that hScrib and PP1γ can exist as a complex in vivo.

### hScrib interacts with PP1γ through a conserved RVxF motif

**Figure 2:**
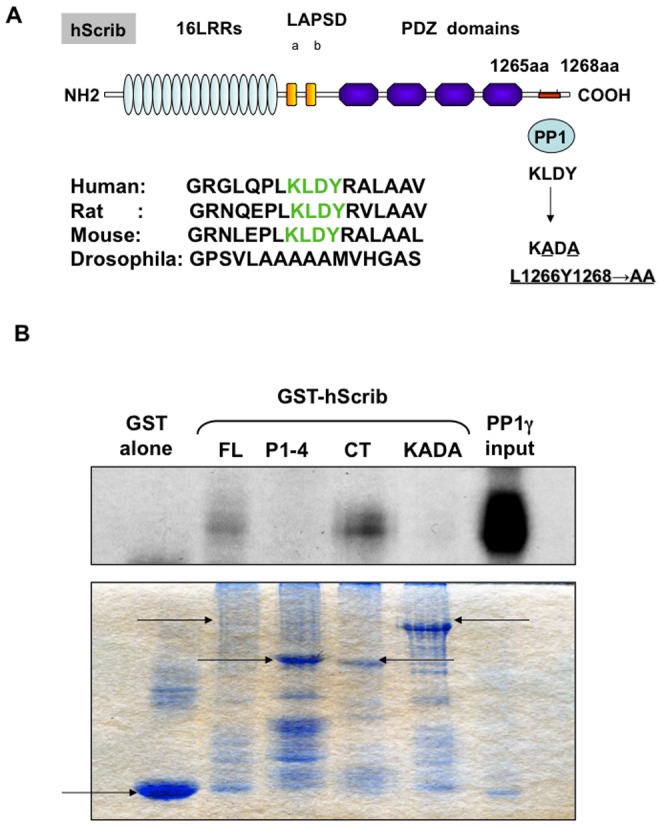
hScrib contains a consensus PP1-binding motif. A) The schematic shows the arrangement of the functional domains on the hScrib protein, highlighting the LRR, LAPSD and PDZ domains. The putative PP1-binding site, the RVXF (the consensus sequence is K/R/H/N/S V/I/L X F/W/Y) motif is also shown, where X is any amino acid. The hScrib mutant in which the PP1-binding site KLDY was mutated to KADA in order to disrupt the interaction with PP1 is shown. A comparison sequence alignment of the region of hScrib containing the PP1-binding motif indicating its absence in Drosophila also shown. B) In vitro translated and radiolabeled PP1γ was incubated with purified full length GST-hScrib fusion protein (FL), GST-hScrib PDZ1-4 (P1-4), GST-hScrib CT (CT), GST-hScrib L1266Y1268→AA (KADA) and GST alone as a control. After extensive washing the bound PP1γ was ascertained by SDS PAGE and autoradiography. The upper panel shows the autoradiograph, with the input of PP1γ also shown for comparison. The lower panel shows the Coomassie stain of the gel showing the levels of GST fusion protein loading, with the arrows indicating the relevant full length fusion proteins.

The PP1 holoenzyme is composed of a catalytic subunit and several regulatory subunits, which target the catalytic subunit to specific subcellular locations. The RVxF motif is a short conserved PP1-binding motif initially identified in previous studies showing that these residues can block the interaction of regulatory subunits with the PP1 catalytic subunit [39]. As shown in Figure 2A, analysis of the hScrib sequence reveals the presence of a putative PP1 binding motif, KLDY (the consensus sequence is: {K/R/H/N}.{S/V/I/L}.X.{ F/W/Y}) [40], [41] spanning residues 1265–1268. This sequence is also highly conserved in mammalian Scrib proteins, but is absent in Drosphila. Based on previous studies, mutation of the L and Y residues would be expected to severely perturb the interaction with PP1 [39]–[42]. To investigate whether this KLDY motif is responsible for the capacity of hScrib to bind to PP1γ, a panel of GST-hScrib fusion proteins consisting of the full length (FL), two truncated proteins encompassing PDZ domains 1–4 (P1-4) and the carboxy terminal third of hScrib (CT), plus a full length hScrib with the KLDY/KADA mutation, were used in pull-down assays with in vitro translated radiolabeled PP1γ. The levels of bound PP1γ were then assessed by SDS PAGE and autoradiography and, as can be seen from Figure 2B, PP1γ binds to the carboxy terminal region of hScrib which contains the predicted PP1 binding motif. Furthermore the KLDY/KADA mutation significantly decreases the capacity of PP1γ to interact with hScrib, confirming that the major site of interaction is through the KLDY consensus motif.

### hScrib and ERK are substrates of PP1γ

**Figure 3:**
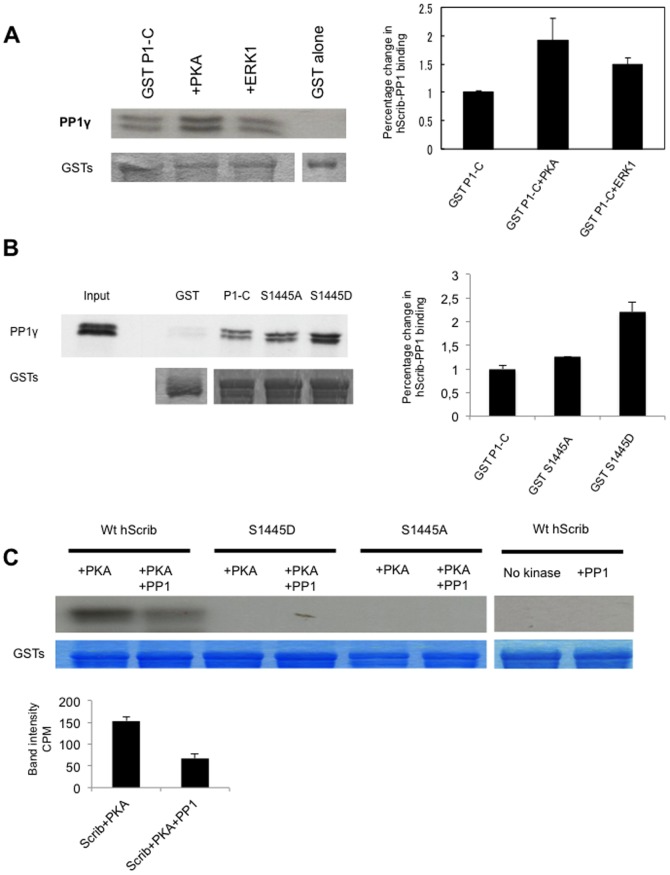
hScrib is a substrate of PP1γ. A) Purified GST-hScrib fusion protein was in vitro phosphorylated with purified PKA or ERK1 as described previously (19) and then incubated with PP1γ for 20 mins at 30°C. Bound PP1γwas detected by western blotting with anti PP1γ antibody. The lower panel shows the ponceau stain of the nitrocellulose, and the upper right panel shows the quantitations from three independent experiments. Note that hScrib phosphorylated by PKA exhibits increased association with PP1γ. B) Purified PP1γ was incubated with purified full length wild type GST-hScrib fusion protein (P1-C), the mutants S1445A, S1445D or GST alone as a control. After extensive washing the bound PP1γ was ascertained by western blotting. The upper panel shows the result of the western blot, with the 20% input of PP1γ also shown for comparison. The lower panel shows the ponceau stain of the nitrocellulose. The histogram shows the quantitation from three independent experiments. C) Purified GST-hScrib wild type and PKA phospho-site mutants of hScrib were in vitro phosphorylated with purified PKA in the presence of radiolabeled ATP as described previously (19) and incubated with PP1γ for 20 mins at 30°C. The remaining level of phosphorylated hScrib was then determined following SDS PAGE and autoradiography. The two right-hand lanes show lack of phosphorylation of hScrib in the absence of PKA, whilst the lower panels show the Coomassie stain of the gel demonstrating equal levels of the GST-hScrib fusion protein throughout. The quantitation of hScrib phosphorylation from three independent experiments is also shown.

We have previously shown that hScrib is a substrate for both PKA and ERK. Furthermore, hScrib can downregulate ERK activation through a direct protein-protein interaction [19], although the precise mechanism by which hScrib can achieve this is still unknown. We therefore wanted to determine whether phosphorylation of hScrib by either PKA or ERK1 could influence the ability of hScrib to interact with PP1γ and, furthermore, whether hScrib itself was a substrate of PP1γ. To do this, purified GST-hScrib fusion protein was subject to phosphorylation by either PKA or ERK1 in the presence of non-radiolabeled ATP, and after extensive washing binding assays were performed using commercially purified PP1γ. The bound protein was then detected by western blotting using anti-PP1γ antibodies. The results in Figure 3A demonstrate a number of important features. In the absence of phosphorylation there is a strong interaction between hScrib and the purified PP1γ, demonstrating that the interaction between hScrib and PP1γ is indeed direct. However, there is also a clear increase in the association between hScrib and PP1γ when hScrib is phosphorylated by PKA, but not when it is phosphorylated by ERK1. We had previously shown that the major PKA phosphorylation site on hScrib was S1445 [19]. Therefore, to further confirm that phosphorylation of hScrib by PKA at S1445 can influence its capacity to interact with PP1γ, we repeated the pull down assays using the phospho-mimic mutation of hScrib, S1445D. As can be seen from Figure 3B, the S1445D mutant exhibits a significantly increased capacity to interact with PP1γ, which is similar to that seen following phosphorylation by PKA. These results demonstrate that phosphorylation of hScrib by PKA at S1445 can indeed increase the ability of hScrib to directly interact with PP1γ.

We then analysed whether hScrib was a potential substrate of PP1γ. Purified GST-hScrib fusion protein was subjected to in vitro phosphorylation with purified PKA and radiolabeled ATP. After extensive washing the radiolabeled hScrib fusion protein was incubated with purified PP1γ, and the amount of phosphorylated protein determined by SDS PAGE and autoradiography. The results obtained in Figure 3C demonstrate that the level of phosphorylated hScrib is decreased following incubation with PP1γ, demonstrating that hScrib is a potential substrate of the phosphatase and, furthermore, that hScrib can directly recruit active PP1γ. Also shown are the non-phosphorylatable mutants of hScrib, confirming the specificity of the phosphorylation reaction.

**Figure 4:**
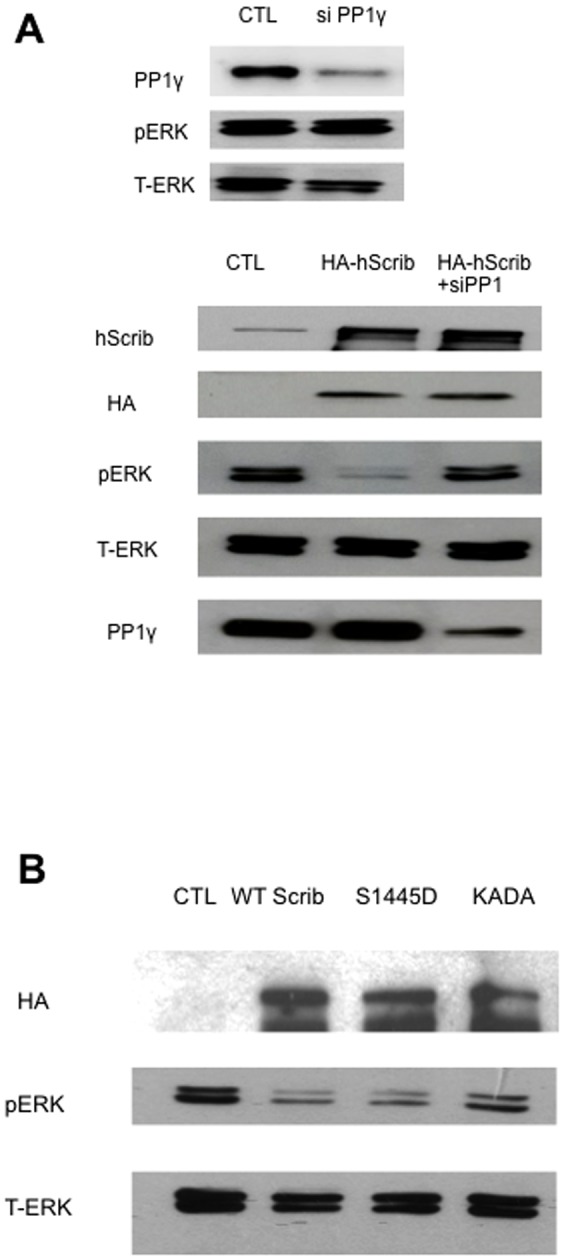
PP1γ is required for hScrib-induced de-phosphorylation of ERK. A) HEK 293 cells were transfected with PP1γ si RNA or si Luc RNA as control (CTL) and after 24 hours were then transfected with a plasmid expressing HA-tagged hScrib. After a further 24 hours the cells were extracted and levels of phospho and total ERK determined by western blot analysis. The upper three panels shows the changes in the ERK profiles when cells were transfected with siRNA PP1γ alone, whilst the lower set of five panels show the effects in the presence of ectopically expressed hScrib. B) HEK 293 cells were transfected with HA-tagged wild type hScrib, or the S1445D and KADA mutants. Total cell extracts were then made after 48 hours and the hScrib, phospho-ERK and total ERK were detected by western blotting.

We then proceeded to determine whether the interaction of hScrib with PP1γ might be involved in the capacity of hScrib to downregulate ERK activation. Cells were transfected with control siRNA against luciferase or against PP1γ, and after 24 hours the cells were then transfected with an hScrib expression plasmid. After a further 24 hours the cells were extracted and the levels of activated phospho-ERK analysed by western blotting. The results obtained are shown in Figure 4A. As can be seen, in the absence of hScrib, siRNA PP1γ has minimal effect on the levels of phospho-ERK (Figure 4A upper three panels). In contrast, overexpression of hScrib significantly reduces the levels of phospho-ERK (Figure 4A lower five panels), and this is in agreement with previous studies [19]. However, the ability of hScrib to down-regulate the levels of phospho-ERK is largely abolished following treatment with siRNA PP1γ, suggesting that this activity of hScrib is in part PP1γ-dependent. To further investigate this, we repeated the assay using the PKA phospho-mimic mutant (S1445D) and the non-PP1γ binding mutant (KADA) of hScrib. After 24 hours the levels of phospho-ERK were analysed by western blotting and the results obtained are shown in Figure 4B. As can be seen the wild type and S1445D mutant of hScrib both strongly inhibit the levels of phospho-ERK, whilst the non-PP1γ binding mutant of hScrib is decreased in this activity. Taken together these results demonstrate that the ability of hScrib to interact with PP1γ correlates with its ability to down-regulate the levels of phospho-ERK.

### Loss of hScrib enhances PP1γ nuclear localization

**Figure 5:**
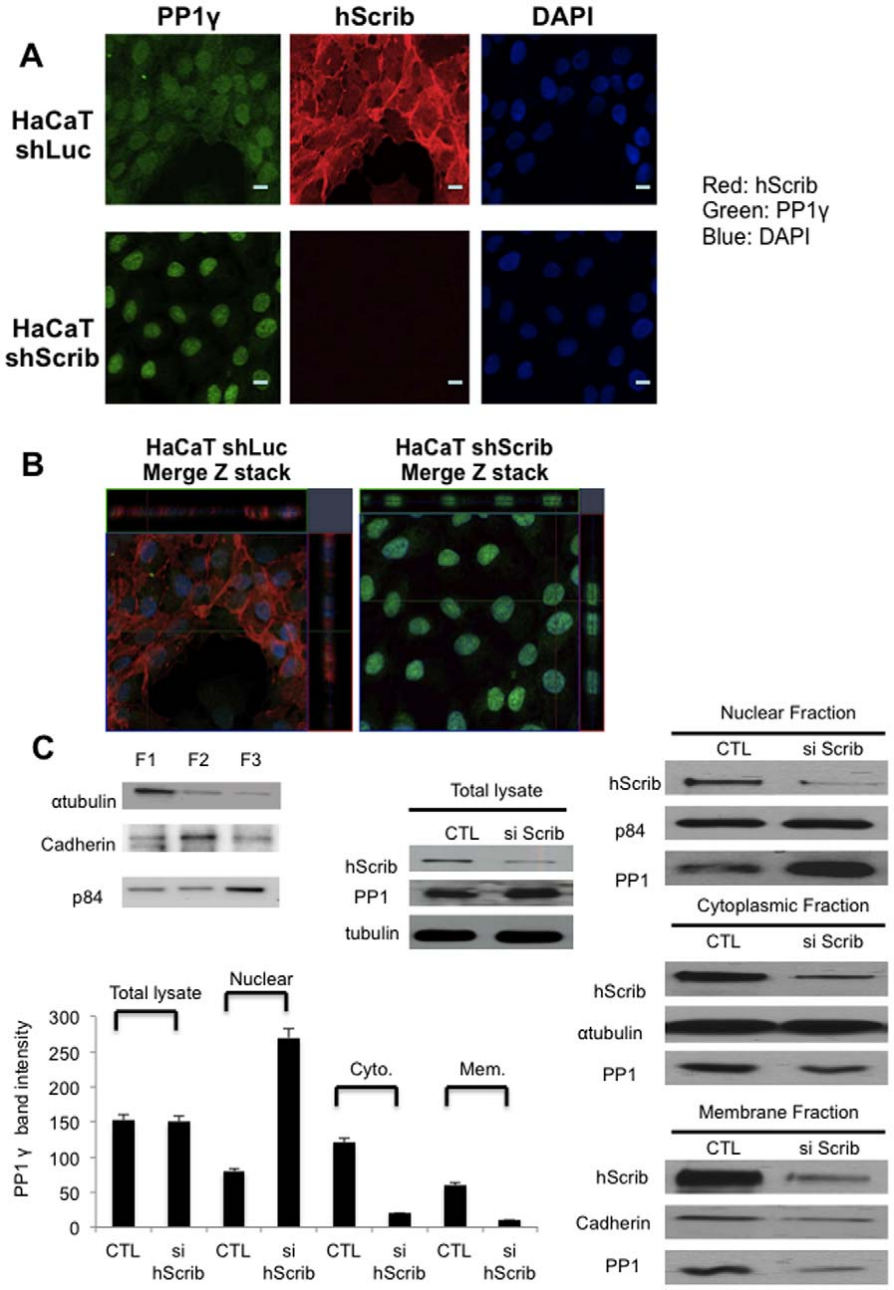
hScrib regulates PP1γ nuclear localization. A) Immunofluorescence analysis of hScrib and PP1γ expression in sh-Luc control HaCaT cells and sh-hScrib knockdown cells. The cells were grown on coverslips and then fixed and double-stained with the anti-hScrib antibody and the anti-PP1γ antibody. Note the significant increase in the levels of nuclear PP1γ in the absence of hScrib expression. B) Z-reconstruction (x-z direction) of a z-stack (15 planes, z-distance 0.2 µm), showing sh-hScrib knockdown cells have enhanced PP1γ localisation into the nucleus. C) HEK 293 cells were transfected with hScrib siRNA and si Luc RNA as control. Cells were either extracted in SDS PAGE sample buffer (Total lysate) or were fractionated into cytoplasmic (F1), membrane (F2) and nuclear (F3) pools (the example shows the integrity of a typical extraction procedure) and then PP1γ was detected by western blotting. p84 was used as a loading control for the nuclear fraction, cadherin was used as a loading control for the membrane fraction and α-tubulin was used as the loading control for the cytoplasmic fraction and total cell extracts. Note the relative increase in nuclear PP1γ following hScrib knockdown but no overall change in total PP1γ levels.

Having found that PP1γ plays a role in hScrib regulation of ERK signaling, we were next interested in determining whether hScrib could also potentially affect PP1γ localisation. Therefore, we first analysed the pattern of PP1γ expression in human keratinocytes after stably silencing hScrib expression in these cells. The distribution of PP1γ in control and shScrib HaCaT cells were analysed by immunofluorescence. The results in Figure 5A and Figure 5B, show that most of the PP1γ localises in the nucleus, although some also co-localises with hScrib at the plasma membrane and within the cytoplasm. More importantly, however, upon loss of hScrib expression there is a significant increase in the amount of nuclear PP1γ, with a corresponding decrease in the cytoplasmic pool. In order to verify these results we also performed a series of transient siRNA experiments, where hScrib levels were ablated in 293 cells, and the levels of PP1γ, both in total cell extracts or in the respective cellular fractions (Fig. 5C), were analysed by western blotting. As can be seen, loss of hScrib resulted in decreases in the cytoplasmic and membrane pools of PP1γ, but a corresponding increase in the amounts of the nuclear form of the protein.

**Figure 6:**
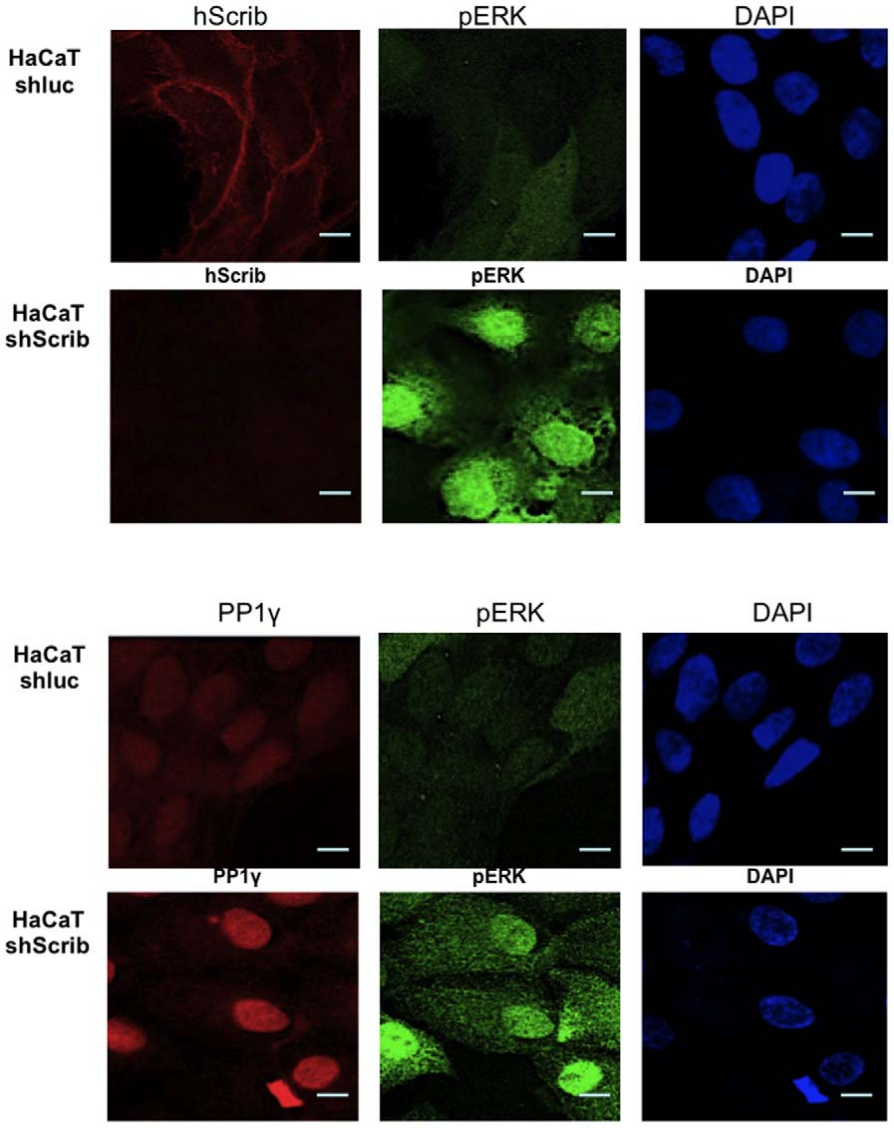
Loss of hScrib results in enhanced nuclear accumulation of both PP1γ and pERK. Control and shScrib HaCaT cells were stained for hScrib, phospho-ERK and PP1γ as indicated.

To investigate the pattern of pERK expression following hScrib depletion we repeated the immunofluorescence assays staining for hScrib, PP1γ and pERK. The results obtained are shown in Figure 6. As can be seen, under conditions of hScrib depletion there is a marked increase in the levels of both nuclear and cytoplasmic pERK, consistent with previous observations [19]. This is also accompanied by an increase in the levels of nuclear PP1γ.

### hScrib tumour suppressor activity requires an intact PP1γ binding motif

**Figure 7:**
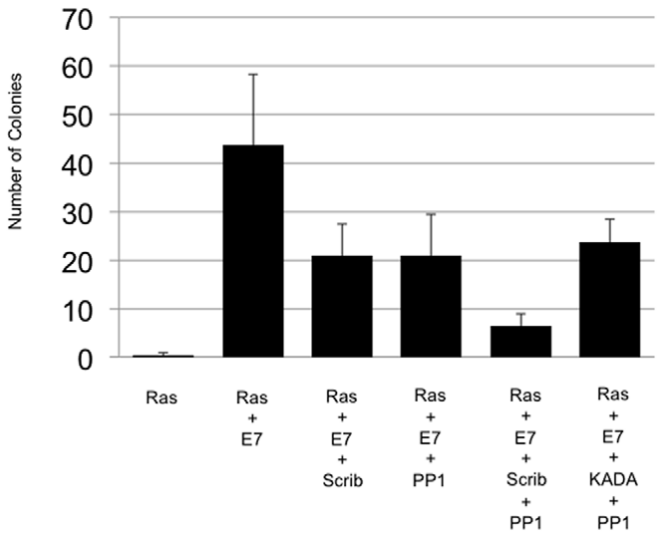
hScrib suppresses HPV-16 E7 and EJ-ras induced transformation in cooperation with PP1γ in a RVxF motif-dependent manner. BRK cells were transfected with EJ-ras alone, HPV-16 E7 plus EJ-ras, HPV-16 E7 plus EJ-ras and wild type hScrib, HPV-16 E7 plus EJ-ras and PP1γ, and HPV-16 E7 plus EJ-ras and wild type hScrib with PP1γ, and HPV-16 E7 plus EJ-ras and PP1γ plus the KADA non-PP1γ binding mutant of hScrib. After three weeks the dishes were fixed and stained and the colonies counted. Results represent the mean number of colonies from 3 independent assays and standard deviations are shown.

We have previously shown that hScrib can suppress cell transformation induced by EJ-ras and Human Papillomavirus (HPV)-16 E7 [19]. To determine whether the interaction between hScrib and PP1γ was physiologically relevant in this context, primary BRK cells were transfected with HPV-16 E7 plus EJ-ras in the presence or absence of the hScrib wild type and KLDY/KADA mutant hScrib expressing plasmids, with or without the PP1γ expression plasmid. After 3 weeks the cells were fixed and stained and the numbers of colonies counted. As can be seen from Figure 7, co-expression of wild type hScrib and PP1γ strongly inhibits the oncogene cooperation between E7 and EJ-ras, whilst the KADA mutant of hScrib is compromised in this activity. These results demonstrate that the hScrib-PP1γ interaction is functionally relevant in an assay of oncogene cooperation.

## Discussion

We have shown previously that hScrib can regulate ERK signaling in two ways. The first involves a direct protein interaction, which is mediated via two KIM binding sites located within hScrib. The second appears to involve the recruitment of a protein phosphatase [19]. In this study we provide evidence that a candidate phosphatase is PP1γ. We have also found that hScrib can control PP1γ sub-cellular localisation, with a loss of hScrib promoting PP1γ nuclear translocation.

Regulation of the ERK signaling cascade can occur at multiple levels and can involve Raf dephosphorylation, MEK1,2 phosphorylation, and also MEK1,2 dephosphorylation [24], [43]–[44]. Furthermore, it has been reported that whilst the kinases in the pathway control signal amplitude, the phosphatase PP2A mediates both signal amplitude and signal duration [32]–[33]. Previous studies have also implicated PP1 in regulating ERK signaling through its ability to dephosphorylate Raf-1 at Ser 259 [45]. Since we have consistently observed that overexpressed hScrib results in a decrease in ERK phosphorylation, we initiated a series of studies to identify the potential phosphatases with which hScrib might interact. Using a proteomic approach we identified PP1γ as a direct interacting partner of hScrib, an interaction that we could confirm both in vitro and in vivo. Analysis of the hScrib amino acid sequence identified a potential site of interaction, KLDY, mutation of which abolished the ability of hScrib to bind PP1γ. Furthermore, this consensus PP1 recognition motif is conserved in mammalian forms of Scrib, but is absent in Drosophila.

We also analysed the effects of PP1γ ablation upon hScrib control of ERK phosphorylation, and found that loss of PP1γ greatly diminished the ability of hScrib to downregulate the levels of phospho-ERK in vivo. Furthermore, we also found that this activity of hScrib was in part dependent upon an intact PP1γ binding site motif. Interestingly, we also noted that the interaction between PP1γ and hScrib was increased following PKA phosphorylation of hScrib, one potential consequence of which is PP1γ-mediated de-phosphorylation of hScrib. Whether this has an important role with respect to other functions of hScrib remains to be determined and is worthy of further study. Taken together these studies demonstrate that hScrib can interact with PP1γ, an activity which appears to play a role in the ability of hScrib to downregulate the ERK signaling pathway. Interestingly, this regulation of ERK by hScrib has many parallels with a recent study showing that hScrib could also regulate Akt signaling [35]. This required hScrib interaction with the phosphatase, PHLPP1, resulting in the de-phosphorylation of Akt. In this case the interaction between hScrib and PHLPP1 requires sequences in the LRR region of hScrib. Thus hScrib could potentially interact simultaneously with multiple protein phosphatases to control diverse signaling pathways. It should also be emphasized that hScrib is a multifunctional protein, and loss of hScrib also results in increased levels of MEK activity, suggesting multiple mechanisms by which hScrib can control ERK signaling [46].

To investigate whether the capacity of hScrib to interact with PP1γ had any physiological relevance, we made use of an oncogene cooperation assay in primary rodent cells. Previous studies had shown that hScrib could suppress cell transformation induced by HPV-16 E7 and EJ-ras in these cells, and that this activity was dependent in part upon the ability of hScrib to interact with ERK [19]. We reasoned that this activity of hScrib might also be influenced by the ability of hScrib to interact with PP1γ. Indeed, both hScrib and PP1γ, either alone or in combination, could dramatically decrease the levels of HPV-16 E7 and EJ-ras induced cell transformation. However, the additive effects upon the levels of cell transformation, seen with the combination of hScrib and PP1γ, was abolished if a mutant hScrib defective in its ability to interact with PP1γ was included in the assay. This demonstrates that, in the context of an oncogene cooperation assay, the ability of hScrib to interact with PP1γ does play a role in the ability of hScrib to suppress cell transformation.

PP1γ has been linked to the regulation of a variety of different cellular processes, including the DNA damage response, nuclear function and diverse aspects of the cell cycle [47]–[52]. One of the important aspects of PP1γ regulation is believed to be related to the control of its nuclear expression, which can be mediated by proteins possessing the consensus RVxF PP1 binding motifs, and which can thereby control the correct cellular localization of PP1 [42], [52]. We therefore investigated whether hScrib might have a similar potential regulatory function with respect to the pattern of PP1γ localization within the cell. This was indeed found to be the case; in two different assay systems we observed that loss of hScrib resulted in an increased nuclear accumulation of PP1γ, with a concomitant decrease in the levels found in membrane and cytoplasmic fractions. Thus hScrib would appear to contribute directly to the regulation of PP1γ expression patterns. Whether this is related to some of hScrib's previously reported pleiotropic effects upon cell proliferation and cell survival remains to be determined. Taken together, these studies have defined PP1γ as a novel interacting partner of hScrib, an interaction which correlates with hScrib downregulation of ERK signaling and suppression of oncogene-induced cell transformation.

## Supporting Information

Figure S1 **Schematic diagram showing the different hScrib expression constructs.** The schematic shows the arrangement of the functional domains on the hScrib protein, highlighting the LRR, and PDZ domains. The putative PP1-binding site, KLDY is also shown in the carboxy terminal third of hScrib. Also summarized are the results on the interaction assays with PP1γ.
(TIF)
