# Supplementary figures and images for: A Novel Interaction between hScrib and PP1γ Downregulates ERK Signaling and Suppresses Oncogene-Induced Cell Transformation
Source: PLoS One. 2013 Jan 24;8(1):e53752. doi: 10.1371/journal.pone.0053752 (PMC3554735; doi:10.1371/journal.pone.0053752)

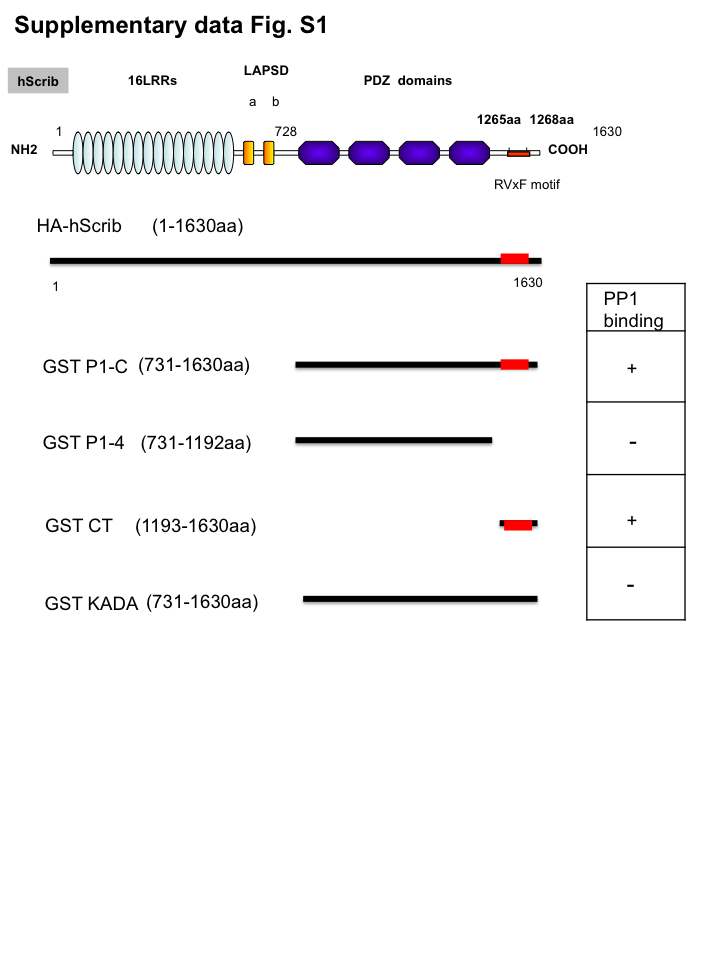

Supplement: Figure S1 — Schematic diagram showing the different hScrib expression constructs. The schematic shows the arrangement of the functional domains on the hScrib protein, highlighting the LRR, and PDZ domains. The putative PP1-binding site, KLDY is also shown in the carboxy terminal third of hScrib. Also summarized are the results on the interaction assays with PP1γ. (TIF) [file pone.0053752.s001.tif]
